# Increased DNA methylation of the *SLC30A8* gene promoter is associated with type 2 diabetes in a Malay population

**DOI:** 10.1186/s13148-015-0049-5

**Published:** 2015-03-18

**Authors:** Norhashimah Abu Seman, Wan Nazaimoon Wan Mohamud, Claes-Göran Östenson, Kerstin Brismar, Harvest F Gu

**Affiliations:** Department of Molecular Medicine and Surgery, Rolf Luft Research Center for Diabetes and Endocrinology, M1:03 Karolinska University Hospital, Karolinska Institutet, Stockholm, Se-17176 Sweden; Cardiovascular, Diabetes and Nutrition Research Centre, Institute for Medical Research, Kuala Lumpur, Malaysia

**Keywords:** DNA methylation, *SLC30A8*, Type 2 diabetes

## Abstract

**Background:**

Recent studies have demonstrated that DNA polymorphisms in the solute carrier family 30 member 8 (*SLC30A8*) gene confer the risk susceptibility to type 2 diabetes (T2D). The present study aimed to analyze DNA methylation levels of this gene in T2D and diabetic nephropathy (DN).

**Results:**

We confirmed the genetic association study of *SLC30A8* in 992 Malay subjects with normal glucose tolerance and T2D patients with and without DN. Genotyping was conducted with TaqMan allelic discrimination. SNP rs11558471(A/G) in the *SLC30A8* gene was strongly associated with T2D (*P* = 0.002, OR = 1.334, 95% CI = 1.110 to 1.602) and moderately associated with DN (*P* = 0.041, OR = 1.399, 95% CI = 1.013 to 1.932). We further performed DNA methylation analysis of six CpG sites in the *SLC30A8* gene promoter with bisulfite pyrosequencing protocol. The average DNA methylation levels of the *SLC30A8* gene in all Malay subjects were at approximately 81.4%. DNA methylation levels of the *SLC30A8* gene in T2D patients were higher compared to non-diabetic subjects (82.9% vs. 80.1%, *P* = 0.014). But no significant difference of DNA methylation levels of the *SLC30A8* gene between T2D patients with and without DN was observed.

**Conclusion:**

The present study thus provides the first evidence that increased DNA methylation of the *SLC30A8* gene promoter is associated with T2D but not DN in a Malay population.

## Background

The solute carrier family 30 member 8 (*SLC30A8*) gene is encoded for a zinc efflux transporter and highly expressed in the pancreas, particularly in alpha, beta, and PP cells of the islets of Langerhans. Functionally, this transporter is essential for zinc flux into beta-cell insulin-secretory granules and the subsequent crystallization of hexameric insulin [1]. Pathological studies have demonstrated that the *SLC30A8* gene expression levels are downregulated in pancreatic islets of diabetic mice [2]. The downregulation of the *SLC30A8* gene expression results in the reduction of insulin content and glucose-inducible insulin secretion [3].

Genome-wide association studies (GWASs) have identified a number of susceptibility variants for type 2 diabetes (T2D). The common alleles of single nucleotide polymorphisms (SNPs), rs13266634(C/T, Arg276Trp), and rs11558471(A/G) in the *SLC30A8* gene are found to confer the risk susceptibility in T2D [4-9]. The genes including *SLC30A8* identified by GWAS, however, can only explain approximately 10% of the overall heritable risk of T2D, which challenges our expectations to translate genetic information into clinical practice [10-12]. The missing information on heritability in T2D includes the impact of rare variants and epigenetic factors. The latter is involved in the complex interplay between genes and the environment [13-15]. To further understand the genetic effects of *SLC30A8*, Flannick et al. have recently conducted a mutation screening study for rare variants in the gene (the minor allele frequency is less 1%) and suggested that the rare variants with loss of function may protect against T2D [16].

Epigenetic factors mainly including DNA methylation changes have been considered to be involved in the pathogenesis of T2D [13-15]. The methylation of the 5′-carbon of cytosine, often in a gene promoter, is a form of epigenetic modification that does not affect the primary DNA sequences but affects secondary interactions that play a critical role in the regulation of gene expression [17,18]. DNA methylation levels are commonly analyzed at clusters of CpG methylation sites in the gene promoter and used for indication of epigenetic effects. We have recently reported that DNA methylation changes of the insulin-like growth factor 1 (*IGF1*) and its binding proteins (*IGFBP1* and *IGFBP7*) are associated with T2D [19-22]. But whether the *SLC30A8* gene is subject to epigenetic effects in T2D is unknown.

Malaysia is a country with a high prevalence of T2D at 14.9% for adults aged 30 years and above according to the latest National Health and Morbidity Surveys in 2006 [23]. Moreover, diabetic nephropathy (DN) is the most common cause of end-stage renal disease (ESRD). In this country, DN contributes to 57% of patients with T2D [24-26]. We have currently collected a cohort of Malay subjects including normal glucose tolerance (NGT) and T2D patients with and without DN for genetic studies. In the present study, we conducted genetic association study and further analyzed DNA methylation alteration of the *SLC30A8* gene in this Malay population. The results may provide useful information to evaluate the genetic and epigenetic effects of the *SCL30A8* gene in T2D and DN.

## Results

### Association of SLC30A8 genetic polymorphisms with T2D and DN

A total of 992 Malay subjects including NGT subjects (*n* = 476) and T2D patients with or without DN (516) were included in the genotyping experiments. Clinical parameters in all Malay subjects selected for genetic association study are summarized in Table 1.

**Table 1:** **Clinical parameters of Malay subjects in genetic association study**

**Group**	**NGT**	**T2D** ^**a**^	**T2D-DN**	**T2D + DN**	***P*** **values** ^**b/c**^
*N* (men/women)	476 (224/252)	516 (260/256)	239 (103/136)	124 (66/58)	
Age (years)	42 ± 15	56 ± 10	55 ± 10	57 ± 10	<0.001/NS
Diabetes duration (years)	-	14 ± 8	13 ± 8	14 ± 7	-/NS
BMI (kg/m^2^)	26 ± 7.0	28 ± 5	29 ± 5	27 ± 5	<0.001/0.059
SBP (mm Hg)	127 ± 19	144 ± 25	139 ± 22	155 ± 31	<0.001/0.001
DBP (mm Hg)	80 ± 11	81 ± 12	80 ± 12	82 ± 13	NS/NS
FPG (mmol/l)	4.87 ± 1.02	5.0 ± 1.22	4.76 ± 1.00	5.6 ± 1.05	0.028/0.035
HbA1c (%)	5.4 ± 0.48	8.1 ± 2.17	7.6 ± 2.0	8.6 ± 2.4	<0.001/<0.001
Serum creatinine (μmol/l)	67.8 ± 33.7	122.3 ± 114.4	91.0 ± 72.9	207.3 ± 178.7	<0.001/<0.001
Urine creatinine (mmol/l)	12.3 ± 7.4	10.0 ± 6.8	11.4 ± 6.2	5.0 ± 3.52	<0.001/<0.001
ACR (mg/mmol)	3.32 ± 16.1	57.3 ± 175.4	1.10 ± 0.819	249.2 ± 311.5	<0.001/<0.001
eGFR (ml/min/1.73 m^2^)	112.9 ± 45.2	71.4 ± 38.2	81.0 ± 29.9	48.3 ± 35.2	<0.001/<0.001

Data are expressed as means ± SD.

*NGT* normal glucose tolerance, *T2D* type 2 diabetes, *DN* diabetic nephropathy, *BMI* body mass index, *SBP* systolic blood pressure, *DBP* diastolic blood pressure, *FPG* fasting plasma glucose, *HbA1c* glycosylated hemoglobin, *ACR* albumin creatinine ratio, *eGFR* estimated glomerular filtration rate.

^a^The patients with microalbuminuria were included.

^b^ *P* values were from tests of NGT vs. all T2D.

^c^ *P* values were from tests of T2D without DN vs. T2D with DN.

**Figure 1:**
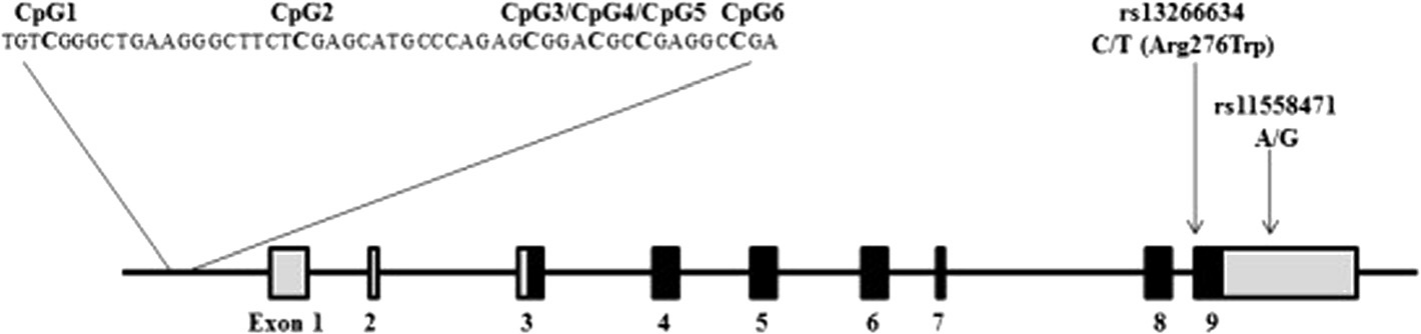
**The CpG sites and single nucleotide polymorphisms in the** ***SLC30A8*** **gene.** The total length of the *SLC30A8* gene is 226,442 bases. RefSeq DNA sequence at NCBI GenBank: NC_000008.10, NC_018919.2, NT_008046.17.

**Figure 2:**
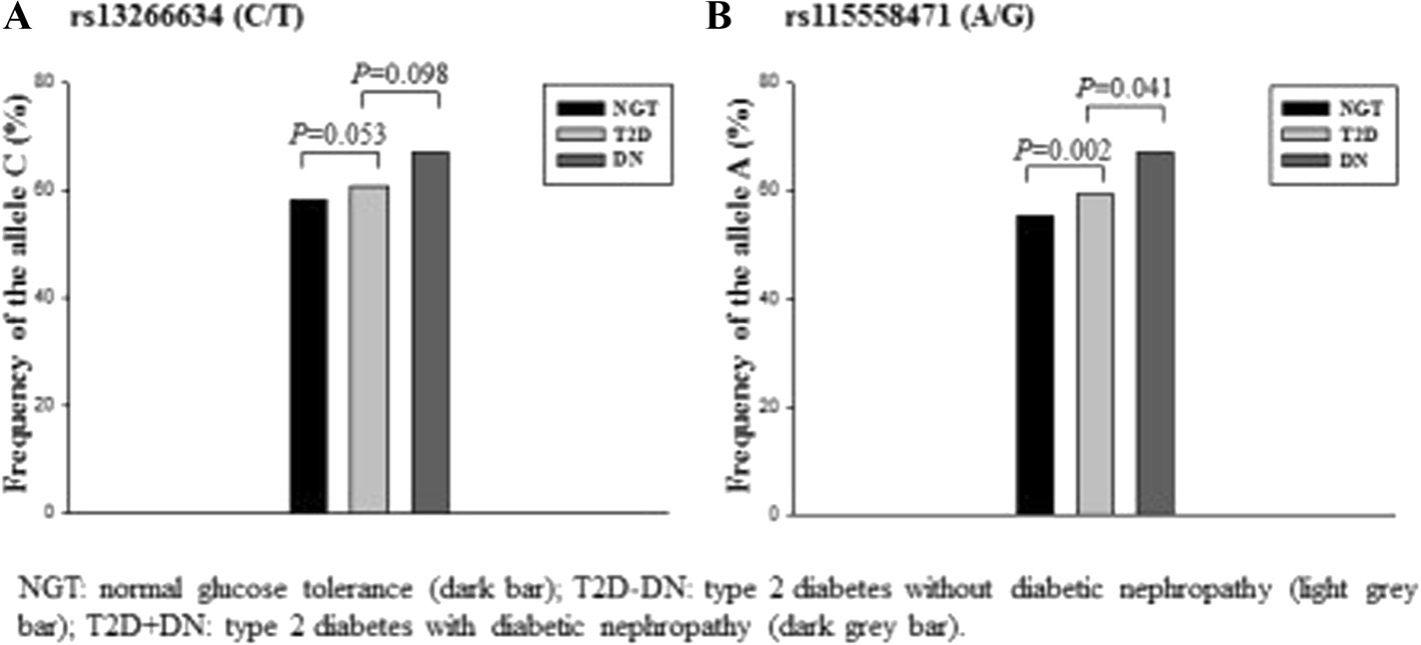
**Risk allele frequencies of the** ***SLC30A8*** **polymorphisms in Malay subjects.** Risk allele frequencies (allele C of rs13266634(C/T) **(A)** and A of rs11558471(A/G) **(B)**) of the *SLC30A8* polymorphisms in Malay subjects with normal glucose tolerance, type 2 diabetes, and diabetic nephropathy. *NGT* normal glucose tolerance (dark bar), *T2D-DN* type 2 diabetes without diabetic nephropathy (light grey bar), and *T2D + DN* type 2 diabetes with diabetic nephropathy (dark grey bar).

The *SLC30A8* gene is located on chromosome 8q24.11. Both SNPs rs13266634(C/T) and rs11558471(A/G) reside within exon 9 of the gene (Figure 1). The first SNP is non-synonymous, in which the amino acid arginine is changed to tryptophan, while the second one is located at 3′-UTR of *SLC30A8.* We genotyped these two SNPs in the Malay subjects with NGT and T2D, and data showed the genotype distributions of these two SNPs in Hardy-Weinberg equilibrium (HWE). Figure 2 illustrates that the frequencies of the major alleles in both SNPs , that is, allele C of rs13266634(C/T) and A of rs11558471(A/G) were increased from NGT to T2D without DN and to T2D with DN. Analyses for single marker association indicated that the A allele of rs11558471(A/G) was strongly associated with T2D (the A allele frequencies between NGT and all T2D were 0.552 vs. 0.620, *P* = 0.002, OR = 1.334, 95% CI = 1.110 to 1.602) and moderately associated with DN (T2D without and with DN, 0.593 vs. 0.671, *P* = 0.041, OR = 1.399, 95% CI = 1.013 to 1.932). The association of SNP rs13266634 (C/T) with T2D and DN was not significant (*P* = 0.053, OR = 1.200, 95% CI = 0.997 to 1.443; and *P* = 0.098, OR = 1.313, 95% CI = 0.950 to 1.815). Information of genotype distribution and allele frequency comparison analyses is summarized in Table 2.

**Table 2:** **Association of the** ***SLC30A8*** **genetic polymorphisms with type 2 diabetes and diabetic nephropathy in a Malay population**

**SNP ID**	**Group**	***N***	**Genotypes**			**RAF**	***P*** **value**	**Odds ratio**
rs13266634			*CC*	*CT*	*TT*			
	NGT	438	153	202	83	0.580	0.053	1.200 (0.997 to 1.443)
	All T2D^a^	506	208	215	83	0.620		
	T2D-DN	236	90	107	39	0.608	0.098	1.313 (0.950 to 1.815)
	T2D + DN	123	59	47	17	0.670		
rs11558471			*AA*	*AG*	*GG*			
	NGT	441	139	209	93	0.552	0.002	1.334 (1.110 to 1.602)
	All T2D^a^	509	204	225	80	0.620		
	T2D-DN	237	87	107	43	0.593	0.041	1.399 (1.013 to 1.932)
	T2D + DN	123	57	51	15	0.671		

Tests were conducted with an additive model.

*NGT* normal glucose tolerance, *T2D* type 2 diabetes, *DN* diabetic nephropathy, *RAF* risk allele frequency.

^a^The patients with microalbuminuria were included.

### Association of SLC30A8 DNA methylation with T2D and DN

**Figure 3:**
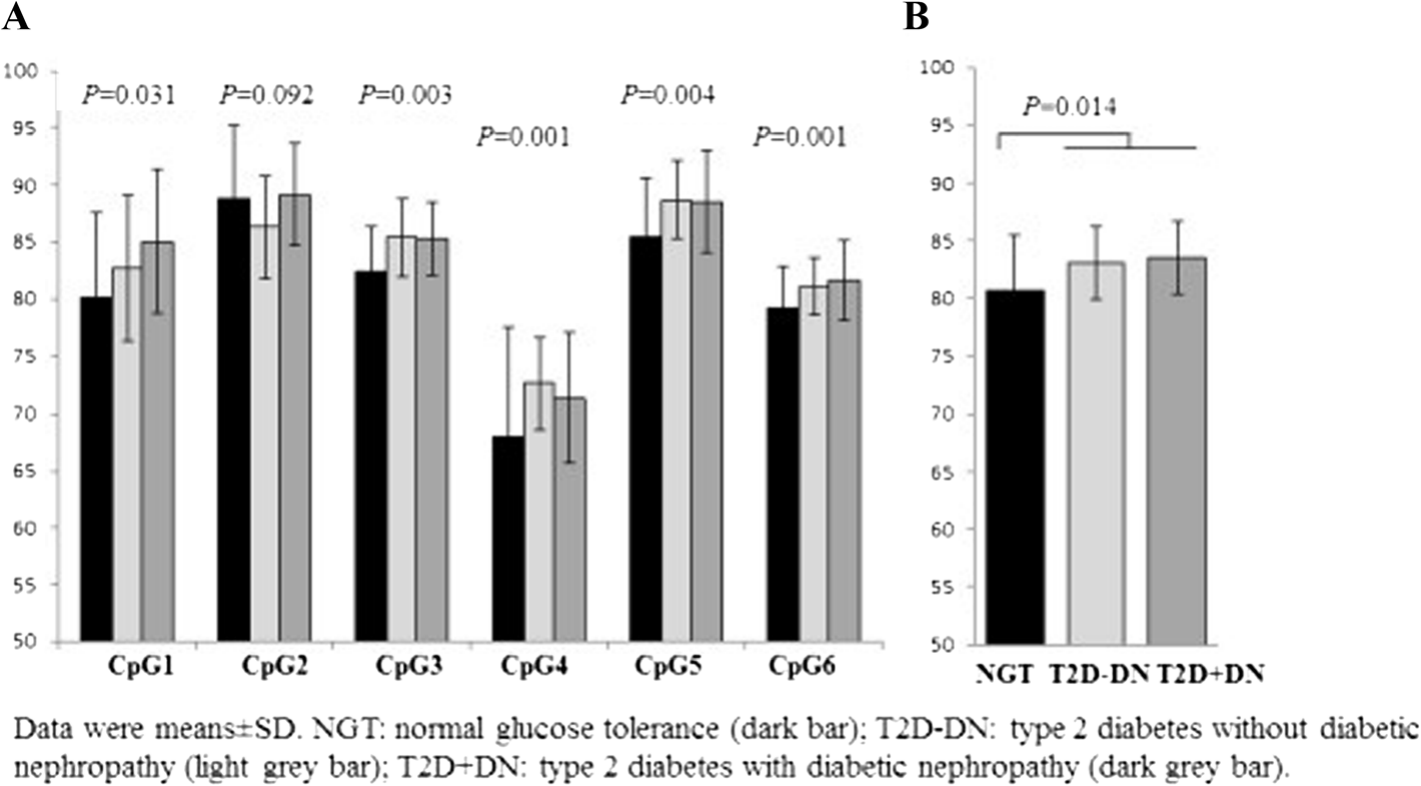
**DNA methylation changes of the** ***SLC30A8*** **gene in Malay subjects.** DNA methylation changes of the *SLC30A8* gene in Malay subjects with normal glucose tolerance, type 2 diabetes, and diabetic nephropathy. DNA methylation levels at five CpG sites of the gene (except CpG2) in T2D patients were found to be higher than those in NGT subjects, respectively **(A)**. Combining all six CpG sites together, total mean values of *SLC30A8* DNA methylation levels were significantly increased in T2D patients compared with NGT subjects **(B)**. Data were means with 95% CI. NGT: normal glucose tolerance (dark bar); T2D-DN: type 2 diabetes without diabetic nephropathy (light grey bar) and T2D + DN: type 2 diabetes with diabetic nephropathy (dark grey bar).

To avoid the error caused by ages, the NGT subjects and T2D patients with age match were selected in DNA methylation analyses (Table 3). In the promoter region of the *SLC30A8* gene, there is a cluster of neighboring six CpG sites (Figure 1). We analyzed *SLC30A8* DNA methylation levels not only at each CpG site but also in total means of all CpG sites. In this Malay cohort, the average DNA methylation levels of all six CpG sites in the *SLC30A8* gene promoter were high (81.4%). Figure 3A demonstrates the DNA methylation patterns of CpG sites in the *SLC30A8* gene promoter region among NGT subjects and T2D patients with and without DN in the Malay cohort. DNA methylation levels at five CpG sites of the gene (except CpG2) in T2D patients were found to be higher than those in NGT subjects, respectively (CpG1 83.9% vs. 81.9%, *P* = 0.031; CpG3 82.1% vs. 84.8%, *P* = 0.003; CpG4 69.6% vs. 66.3%, *P* = 0.001; CpG5 86.2% vs. 83.7%, *P* = 0.004; and CpG6 79.8% vs. 78.1%, *P* = 0.001) (Figure 3A). Combining all six CpG sites together, total mean values of *SLC30A8* DNA methylation levels were significantly increased in T2D patients compared with NGT subjects (82.9%, 95% CI = 79.2% to 80.5% vs. 80.1%, 95% CI = 75.4% to 78.6%, *P* = 0.014) (Figure 3B). A linear regression model was used to estimate the association between SLC30A8 DNA methylation levels in blood and phenotypes of T2D, and no significant association was found. Furthermore, the analyses of *SLC30A8* DNA methylation levels between the patients with and without DN or according to the genotypes of SNPs rs13266634(C/T) and rs11558471(A/G) were done and no significant difference of the levels was found.

**Table 3:** **Clinical parameters of Malay subjects in epigenetic study**

**Group**	**NGT**	**T2D** ^**a**^	**T2D-DN**	**T2D + DN**	***P*** **values** ^**b/c**^
*N* (men/women)	27 (19/8)	161 (81/80)	109 (49/60)	43 (25/18)	
Age (years)	57 ± 9	56 ± 9	55 ± 10	56 ± 10	NS/NS
Diabetes duration (years)	-	12 ± 7	11 ± 8	11 ± 7	-/NS
BMI (kg/m^2^)	24 ± 4.0	28 ± 5	29 ± 5	27 ± 5	<0.001/NS
SBP (mm Hg)	132 ± 18	139 ± 24	135 ± 20	150 ± 33	NS/NS
DBP (mm Hg)	84 ± 14	80 ± 12	80 ± 12	83 ± 15	NS/NS
HbA1c (%)	5.7 ± 1.0	8.2 ± 2.0	8.1 ± 2.0	8.6 ± 2.4	<0.001/NS
Serum creatinine (μmol/l)	70.6 ± 17.1	126.4 ± 119.5	92.7 ± 72.9	215.8 ± 183.0	<0.005/<0.001
Urine creatinine (mmol/l)	12.3 ± 7.4	8.1 ± 6.8	9.7 ± 6.2	5.0 ± 3.52	<0.001/<0.001
ACR (mg/mmol)	0.86 ± 0.75	46.2 ± 127.1	1.19 ± 0.84	195.1 ± 206.1	<0.001/<0.001
eGFR (ml/min/1.73 m^2^)	102.8 ± 28.6	67.1 ± 28.0	76.5 ± 23.4	44.1 ± 26.0	<0.001/<0.001
SLC30A8 DNA methylation levels (95% CI)	80.1% (79.4 to 81.4)	82.9% (81.2 to 83.5)	82.7% (80.9 to 82.9)	83.6% (79.8 to 84.4)	0.014/0.632

Data are expressed as means ± SD or 95% CI.

*NGT* normal glucose tolerance, *T2D* type 2 diabetes, *DN* diabetic nephropathy, *BMI* body mass index, *SBP* systolic blood pressure, *DBP* diastolic blood pressure, *FPG* fasting plasma glucose, *HbA1c* glycosylated hemoglobin, *ACR* albumin creatinine ratio, *eGFR* estimated glomerular filtration rate.

^a^The patients with microalbuminuria were included.

^b^ *P* values were from tests of NGT vs. all T2D.

^c^ *P* values were from tests of T2D without DN vs. T2D with DN.

## Discussion

We have investigated DNA methylation levels of the *SLC30A8* gene in a Malay population. Our main findings include: first, a cluster of CpG sites in the promoter region of the *SLC30A8* gene is hypermethylated. Second, increased DNA methylation levels of this gene are associated with T2D but not with DN.

DNA methylation analysis can be performed in the scales of global genome or specific gene region and in peripheral blood with mixed cell types or specific tissues [27]. Dayeh et al. have recently performed human pancreatic islet tissue-specific DNA methylation analyses at the genomic regions of SNPs that are predicted to be associated with T2D by GWAS [28]. In the *SLC30A8* gene, three CpG sites are located nearby SNP rs13266634. According to the genotypes of this polymorphism, DNA methylation changes at one of CpG sites are found to be significantly different. However, the DNA methylation levels at this CpG site are very low (<5%). Hall E et al. have further investigated the effects of palmitate on genome-wide mRNA expression and DNA methylation patterns in human pancreatic islets. With the palmitate treatment, the *SLC30A8* mRNA expression levels in human pancreatic islets are found to be decreased. But whether DNA methylation levels of this gene are affected by palmitate is unclear [29]. A recent report has indicated that both approaches of whole-blood DNA methylation profiling and adipose tissue-specific methylation analysis for study of epigenetic changes are related to body mass index (BMI) and suggested that the analysis of blood DNA methylation is worthwhile and can reflect changes in relevant tissues for a phenotype [30]. The present study is a clinical observation. Although we have no tissue sample of pancreatic islets available for analysis, epigenetic study with blood samples is clinically accessible. Our findings are unlikely false positive because the average of DNA methylation of the *SLC30A8* gene is high. We have analyzed the *SLC30A8* gene DNA methylation levels according to gender and smoking factors; no significant change of the levels between NGT and T2D was found. Furthermore, the increased DNA methylation of the *SLC30A8* gene in T2D is consistent with the downregulation of the gene expression in pancreatic islets of diabetic animals as previously reported [2,3]. Except age, gender, and smoking, the confounding factor of ethnic group has been taken into our study. Malaysian populations include Malays, Chinese, Indians, and indigenous Sabahans and Sarawakians. To avoid the error caused by this confounding factor, only Malay subjects are selected for this study. However, the replication study in other ethnic groups or populations may be necessary to ascertain the *SLC30A8* epigenetic effect in T2D.

In the recent years, several groups including ours have begun to investigate the epigenetic effects in T2D. From our studies [19-22] and other reports [27-30], we have learnt that DNA methylation levels between non-diabetic control subjects and T2D patients differ significantly but the difference is small (often around 2%). The standard deviation of detected DNA methylation values is generally much less compared with the data from serum or plasma protein analyses. Compared with an epigenetic study in tumor, it may be more difficult to perform the same study in T2D. There are a number of genes contributing towards genetic and epigenetic effects to the disease, and the contribution of each gene may be minor. In this case, it is of importance to analyze the accumulating genetic and epigenetic effects in T2D. Data from the present study demonstrate that the *SLC30A8* gene DNA methylation is high and implicate that the association of increased *SLC30A8* DNA methylation levels with T2D should be included into the accumulating analyses.

Compared with genetic study of T2D, the progress of genetic study of DN has been slowed down mainly due to the difficulty of collection and characterization of subjects [31]. Although genetic association studies of the *SLC30A8* genetic polymorphisms with T2D are well documented [4-9], no association of the genetic polymorphisms with DN has been reported. In the present study, we have replicated the association of the *SLC30A8* genetic polymorphisms with T2D and also shown a moderate association between SNP rs1155847(A/G) in the gene and DN. The data provide the basic information for further epigenetic analysis of the *SLC30A8* gene in the present study. However, the sample sizes of cases (T2D patients with DN) and controls (T2D without DN) are relatively small. Additional investigation with large cohorts is necessary to confirm the association of the *SLC30A8* genetic polymorphisms with DN.

## Conclusion

In conclusion, the present study provides the first evidence that increased DNA methylation of the *SLC30A8* gene is associated with T2D but not DN in a Malay population.

## Methods

### Subjects

Malaysia is a country with multi-cultures and multi-ethnic populations. We collected the subjects with NGT and T2D from the collaborating centers all over Malaysia. The ethnic distribution of our cohort was 67.6% Malays, 15.3% Indians, 14.8% Chinese, and 2.3% indigenous Sabahans and Sarawakians. To avoid the error caused by ethnic stratification, Indian, Chinese, and indigenous Sabahans and Sarawakians were excluded in the present study. A total of 950 Malay subjects with NGT (*n* = 441) and T2D (509) were included in the genetic association study. The patients with T2D were diagnosed based on medical history by the medical officer or by oral glucose tolerance test (OGTT). All diagnoses were done based on World Health Organization (WHO) criteria [32]. The patients with T2D and normoalbuminuria (ACR <3.5 mg/mmol) were considered as control subjects without DN, while the patients with macroalbuminuria (ACR ≥35 mg/mmol) and ESRD who needed dialysis were included as the cases with DN. The diagnoses of DN were based on urine albumin-to-creatinine ratio (ACR) suggested by ADA [33]. There were 131 T2D patients with microalbuminuria (ACR 3.5 to 35 mg/mmol). Finally, 237 patients without DN and 123 patients with DN were included in the study. Clinical characteristics in all Malay subjects selected for genetic association study and epigenetic analysis are summarized in Tables 1 and 3, respectively.

The informed consent was given from all subjects, and the study was approved by the local ethical committees. All subjects need to answer a questionnaire and underwent a medical examination guided by the research staff. Data and material transfer agreement from the Institute for Medical Research, Malaysia to Karolinska Institutet, Sweden was completed prior to the study.

### Genomic DNA extraction

Genomic DNA extraction was isolated from fresh peripheral blood samples using a DNeasy blood and tissue extraction kit according to the manufacturer’s protocol (Qiagen, Hilden, Germany) and quantified using a spectrophotometry (Biophotometer Plus, Eppendorf, Germany).

### SNP selection and genotyping

Based upon information from the recent genetic association studies, two SNPs, that is, rs13266634(Arg276Trp C/T) and rs11558471(A/G) in the *SLC30A8* gene, were selected for our study [4-9]. The first SNP is non-synonymous, in which the amino acid arginine is changed to tryptophan, while the second one is a synonymous SNP at the 3′-UTR. The *SLC30A8* gene structure and location of these two polymorphisms are shown in Figure 1. Genotyping these two SNPs were done using TaqMan SNP genotyping assays purchased from Applied Biosystems (Applied Biosystems, Foster City, USA). Genotyping experiments were performed in ABI 7300 sequence detector with a Taqman allelic discrimination protocol (Applied Biosystems). DNA samples were distributed randomly across plates with cases and controls for genotyping quality control. All PCR reactions were run in 20 μl volumes using 10 to 20 ng genomic DNA. Millipore water was used as negative controls (blanks) on each plate.

### Bisulfite treatment and pyrosequencing

Epigenetic analysis was performed by bisulfite pyrosequencing, which is a sensitive and accurate protocol [34,35]. DNA was treated with sodium bisulfite using EpiTect Bisulfite kit (Qiagen) and cleanup of bisulfite-converted DNA was done. PCR amplification was then carried out using PyroMark CpG assay (Qiagen) and PyroMark Gold Q96 Reagent kits (Qiagen) in a PyroMark Q96 system (Biotage AB, Uppsala Sweden). PyroMark PCR master mix includes HotStarTaq DNA polymerase and optimized PyroMark reaction buffer containing 3 mM MgCl^2^ and dNTPs, 10x CoralLoad Concentrate, 5x Q-Solution, 25 mM MgCl2, and RNase-free water. The PCR amplicon covers the sequence in human chromosome 8: 117962434 to 117962479 (version 37.56). There are four CpG sites in the *SLC30A8* gene promoter region as indicated with the bold letter ‘**C**’ and recorded as CpG1-4 (Figure 1). Finally, methylation levels of these CpG sites were detected by using the PyroMark Gold 96 Reagent kit (Qiagen) and a PyroMark Q96 ID pyrosequencing system (Biotage). The unmethylated and unconverted DNA samples (Qiagen) were used for control of conversion efficiency in bisulfite treatment and accuracy in methylation analyses. PyroQ-CpG software (Biotage) was used for methylation data analysis.

### Statistical analyses

Allele frequency and genotype distribution of the studied SNPs were tested for HWE using the χ^2^ statistic. For difference between NGT subjects and T2D patients, two models were tested comparing either allele frequencies in 2 × 2 contingency tables (dominant) or genotypes in 3 × 2 contingency tables (additive). Odd ratios (OD) and 95% confidence intervals (CI) were calculated to test the relative risk for association. Statistical power for genetic association study was calculated by using the software of PowerSampleSize (PS version 2.1.31). Since the major alleles of the studied *SLC30A8* polymorphisms were risk for T2D, and their frequencies were high (55% to 67%), the sample sizes of cases and controls included in our studies were sufficient to detect an association with T2D at 85% powers. But the power to detect the association with DN is low (approximately 55%). Tests for comparison of continuous variables between groups were assessed by unpaired *t*-test or one-way ANOVA followed with Tukey’s *post hoc* test. Data in non-normally distributed traits were transformed to the natural logarithm for obtaining a normal distribution before performing statistical analysis. To estimate the association between SLC30A8 DNA methylation levels in blood and phenotypes of T2D, the linear regression model was used. Data were given as the means ± SD. *P* value less than 0.05 was considered significant. Statistical calculations were performed by using PASW Statistic Base 18 (SPSS Inc, Chicago, IL, USA).

## Abbreviations

T2D: Type 2 diabetes
SLC30A8: Salute carrier family 30
SNP: Single nucleotide polymorphism
